# Information on dysregulation of microRNA in placenta linked to preeclampsia

**DOI:** 10.6026/97320630017240

**Published:** 2021-01-31

**Authors:** Abdifatah Mohamed Nuh, Yan You, Min Ma

**Affiliations:** 1 Department of Obstetrics, Affiliated Hospital of Yangzhou University, Yangzhou, Jiangsu Province, 225000, China; 2 Yangzhou University Medical College, Yangzhou, Jiangsu Province, 225000, China

**Keywords:** MicroRNAs, preeclampsia, pregnancy, placenta, trophoblast

## Abstract

MicroRNAs are single-stranded, non-coding RNA molecules, regulate gene expression at the post-transcriptional level. They are expressed in the human body and have a significant impact on the different processes of pathological illness. A developing placenta undergoes a series of stages after successful fertilization, such as cell division, migration, adhesion, apoptosis, and angiogenesis. MicroRNAs dysregulation in placenta has been linked to pregnancy-related complications such as preeclampsia. Therefore, it is of interest to document known information (list of microRNA) on this issue in the development of biological tools for diagnosis, treatment and prevention of the disease.

## Background

Preeclampsia (PE) is a new onset of elevated blood pressure after 20 weeks of gestation in a pregnant mother or 6 weeks postpartum, clinically characterized by maternal heterogeneous systemic conditions of new-onset hypertension and proteinuria [1]. Extravillous cytotrophoblasts of foetal origin invade the uterine spiral arteries of the decidua and myometrium in the early stages of normal placental development. The endothelial layer of the maternal spiral arteries is replaced by these intrusive cytotrophoblasts, transforming them from small high-resistance vessels into large capacitance vessels capable of providing sufficient placental perfusion to nourish the foetus. In PE, this transformation is incomplete [2]. The pathogenesis of PE follows into two main stages; (a) abnormal placentation resulting from inadequate placental cytotrophoblast invasion and spiral artery remodeling dysfunction in the first trimester and (b) maternal systemic syndrome in second and third trimesters or within the postpartum period [3,4] Abnormal placentation is contributed by the failure of trophoblast invasion, hypoxia at the Maternal-Fetal Interface, and the dysfunction of released Reactive Oxygen Species and the Uterine Natural Killer Cells [5-7]. Extensive studies further confirmed the involvement of epigenetic gene alternation in the development of PE [8]. Epigenetic regulators are molecules that control gene expression (degrades mRNA/blocks translation) without altering the target DNA sequence [9]. Classic examples of these molecules are the miRNAs [10]. Differential miRNA expression analysis identified miRNA-210 dysregulation in placentae from pregnancies complicated with PE [11]. Excess outflow of placental soluble toxic factors in the maternal circulation results in endothelial dysfunction, inflammation, and maternal systemic disease. However, it is uncertain that whether the above mentioned pathways are interrelated, or act independently [12,13]. For the last decade, extensive studies have primarily focused on placental miRNAs, but also raised several concerns about the extent of their biological action [14]. This review aims to provide an updated summary of placenta-specific miRNA dysregulation in preeclampsia.

## An overview on microRNA:

Unlike other traditional gene products, microRNAs are small molecules derived from original genes through the lineage of all multicellular animals and plants [15]. The synthesis of miRNA begins initially with the development of primary transcripts known as pri-miRNAs, Transcribed by RNA polymerase II and RNA polymerase III [16]. The initial loop structure is recognized and processed by the Drosha and double-stranded RNA binding protein DGCR8, the protein complex of ribonuclease to form precursor miRNA (pre-miRNA)[17]. The pre-miRNA is exported from the nucleus to cytoplasm by exportin 5 that uses the energy supplied by the GTP complex Ran- (ras-related nuclear protein) [18]. In the cytoplasm, under the action of Dicer, the loops and part of the stem structure of pre-miRNA are cleaved which results in a double-stranded RNA (dsRNA) molecule with 19-25 nucleotides [19]. They are not translated into proteins and hence form total cellular RNAs [20]. In the cytoplasm, further events involving alignment and arrangements occur to form short 18-25 nucleotide segments called microRNAs [21]. The fundamental function of miRNA is to regulate gene expression, both at the transcription and post-transcriptional stages [22,23]. It influences several biological processes, including cell growth, differentiation, cell cycle, metabolism, and apoptosis [24]. Liver-specific miRNA-122, for example, is involved in controlling genes related to cholesterol and lipid metabolism and is also targets the hepatitis C virus [25]. Research into two miRNA families, i.e. miRNA-1 and miRNA-133, revealed their expression in the heart that regulates heart development and related diseases [26,27]. miRNA binds to its target genes based on complementary base pairing, induces cleavage or repression (reviewed in [28-30].

## Placental development and pathogenesis of preeclampsia:

Upon successful fertilization, several maternal physiological modifications, such as hemodynamic and cardiovascular changes, are linked with the normal pregnancy to fulfill the oxygen and nutrient requirements of the developing fetus [31]. Among the fundamental changes include; systemic vasodilation, decreased vascular resistance, increased blood volume and cardiac output, and a slight decrease in blood pressure (BP)[32,33]. The mechanism behind these changes includes increase in different vasodilator substances extracted from endothelium and redistribution of blood flow in various maternal tissues and organs [34,35]. Subsequently, 6 to 8% of pregnant women may have hypertension in pregnancy (HTN-Preg) [36]. These kinds of cardiovascular pregnant related disorder may be manifested in one of four forms: chronic HTN that predates pregnancy, preeclampsia (PE)-eclampsia, chronic HTN with superimposed PE, and nonproteinuric gestational HTN [37]. Other key change to ensure proper placenta and vascular development is angiogenesis, establishment of vasculogenesis, and trophoblast-mediated remodeling [38]. Vasculogenesis is characterised as the growth of de novo vessels from pluripotent mesenchymal stem cells, while angiogenesis is the development of new blood vessels by branching from pre-existing ones [39]. Placental extravillous trophoblasts (EVTs) invade up to one-third of the myometrium in the maternal decidua during the first trimester and shift the spiral arteries from low-capacity high-resistance vessels to high-capacity low-resistance vessels, thus ensuring adequate blood supply to the developing fetus [40]. Inadequate placenta establishment is believed to be initiated by major histocompatibility complex (MHC) molecules, natural killer (NK) cells, abnormal expression of cytokines, and macrophages [41]. Abnormal integrin and matrix metalloproteinase (MMP) expression may also contribute to shallow trophoblastic invasion, reduced remodeling of the extracellular matrix (ECM), and spiral arteries [42]. After the 20th week of pregnancy, some women develop new-onset HTN (systolic pressure ≥140 mmHg and/or diastolic pressure ≥90 mmHg), proteinuria, and may be associated with edema and increased platelet aggregation [43]. These clinical-pathological characteristics are referred to as placenta eclampsia. In more severe cases, HELLP syndrome is a syndrome with clinical manifestations clustered together, manifested as hemolysis, elevated liver enzymes, and reduced platelet counts [44]. PE may progress to eclampsia, characterized by extreme HTN and convulsions that may culminate in coma and death, if untreated, causing an estimated 14% of maternal deaths associated with pregnancy [45]. On the fetus side, PE may cause intrauterine growth restriction (IUGR) and preterm birth, causing ~13% of premature births in the United States [46]. The incidence and complications of PE are significant in developing countries where the prevalence of HTN-Preg is greater and the rates of maternal mortality and preterm births are higher than those in developed countries [47].

## miRNAs in the placenta:

During pregnancy, microRNAs are abundantly expressed in the placenta, demonstrated by Liang et al., Meng Cai et al. [48,49]. The main source of placenta miRNAs is the villous trophoblasts [50]. Most miRNAs in the placenta are expressed in clusters. The common gene clusters found abundantly in human placenta trophoblasts are chromosome 19, C19MC which spans about 100 kb of genomic DNA and harbors 46 intronic miRNA genes that express 58 miRNA species [51]. For C19MC miRNAs paternally inherited allele is only expressed in the placenta. C19MC miRNAs have been found as early as week 5 of pregnancy and their expression shows a correlation with the pregnancy stages [52]. For example, Hromadnikova et al. reported the upregulation of circulating C19MC miRNAs (miRNA-516-5p, miRNA-517*, miRNA-520a*, miRNA-525, and miRNA526a) in patients with PE [53]. High levels of expression cluster have been found to play a significant role in providing resistance to infections by viruses [54]. These miRNAs function by inducing autophagy in placental cells. Interestingly, overexpression of the C19MC cluster also confers viral resistance to non-placental cells which strongly indicates that this cluster is important to attenuate invading viral pathogens. Placenta-specific miRNA expression and their association in various pregnancy complications are reviewed in [55,56].

## Dysregulation of miRNAs in the placenta:

### Evidence of upregulation of placenta-specific miRNAs:

Many local complications linked to pregnancy develop at the placenta site. Knowing that miRNAs play an important role in several processes, an aberrant placental expression of miRNAs would correlate with several pathological diseases including PE. A study conducted by Jairajpuri et al [57], for example, evaluated the expression of miRNAs in the plasma of 15 women with PE. They reported the elevated expression of several miRNAs, namely, miRNA-215, miRNA-155, miRNA-650, miRNA-210, miRNA-21, miRNA-518b, and miRNA-29a. Hypoxia-induced trophoblast cell lines showed increased expression of miR-210 in the placentas of PE patients [58]. Similarly, another study revealed the up-regulation of miR-517a/b (p = 0.0085) and miR-517c (p = 0.0043) in preeclamptic placentas [59]. Recent experimental data indicate that these miRNAs regulate angiogenesis, trophoblast proliferation, and immune tolerance (Key processes in PE) [52]. Angiogenesis is known to play a pivotal role in PE pathogenesis and several miRNAs (angiomiRNAs) are known to alter angiogenic pathways. For example, overexpression of miRNA-16 and miRNA-29 inhibit vascular endothelial growth factor (VEGF)-A, leading to the inhibition of migration of human umbilical vein endothelial cells (HUVECs) [60]. When miRNA-494 is overexpressed, it arrests the G1/S transition by targeting cyclin-dependant kinase 1 (CDK1) and cyclin D1 (CCND1) [61]. The supernatant from miRNA-494 overexpressing dMSCs impairs HUVEC capillary formation by suppressing VEGF. Furthermore, cytotrophoblastic overexpression of miRNA-17, miRNA-20a, and miRNA-20b causes defective cytotrophoblast migration and spiral artery remodeling by targeting Ephrin B2 and Ephrin B4 [62]. Failure of trophoblast invasion leads to defects in spiral artery remodeling, a hallmark of PE pregnancies [63]. Another well-studied cluster is the miRNA-17-92, it is associated with the regulation and differentiation of primary human trophoblasts [64]. An experimental study on mice showed that the deletion of miRNA-17-92 cluster gives rise to smaller pups that eventually die at birth [65]. Dysregulation of miRNA- 17-92 cluster, miRNA-146a, miRNA-155, and miRNA-223 has been linked to PE and they are also associated with many immune cells such as macrophages, dendritic cells, and Tregs function [66]. miRNA-126 also modulates innate immune responses in plasmacytoid DCs and decidual mesenchymal cells [67, [68]. A complex interplay between MCs and miRNA-181a has been established and miRNA-181a was found to be upregulated in MSCs of severe PE compared to normal patients [69]. Moreover, through increasing NK cell-mediated cytolysis, miRNA-152 plays a role as an immune response enhancer [70]. The rest of the placental microRNAs are expressed differentially according to gestational age [71]. The exact control mechanism is not entirely understood. Hypoxia, however, seems to be a big factor in regulating their placental activity [72,73].

### Evidence of downregulation of some placenta-specific miRNAs:

Jairajpuri et al have reported that in the placental sample taken from a preeclampsia mother, miRNA-18a, miRNA-19b1, miRNA-144, miRNA-15b are downregulated [57]. Comparing to the non-preeclampsia women, miRNA-144 was significantly elevated p<0.005 in a recent study done by Li, H et al [74]. In this study, miR-144 was chosen for its regulating function in ischemia and hypoxia. The role of miRNAs is to regulate the CUGBP2-COX-2 pathway [75], an important protection against simulated ischemia/reperfusion-induced cardiomyocyte death. Subsequent studies have confirmed the downregulation of miR-144 and miR-223 in PE [76 - Check with Author] 77]. Low PE miR-34a expression was reported to be linked with SERPINA3 expression, plays role in trophoblast invasion [78]. MiR-126 downregulation adds to endothelial dysfunction [79]. Interestingly, based on the population under study miRNA showed varied expression patterns (see Table 1 in PDF) from [80],[85-131]. During the early onset of PE, miR-26a-5p, miR-103a-3p, and miR-145-5p have been found to be downregulated and may be associated with long-term cardiovascular diseases [81]. It has been shown to increase trophoblast cell apoptosis by downregulation of miR-378a-5p and miR-376c [82]. Other important miRNAs that are downregulated in PE include miRNA-1, 126, 139-5p, 150, and 576c [77,79, 83,84].

## Conclusion:

Several studies showed that the main cause of PE is placental dysfunction. However, the pathophysiology of this condition is also attributed to genetic variables, immune factors, and systemic inflammation. So far the existence of many miRNAs and their role in disease progression has been confirmed by differential gene expression analysis, but the exact mechanism of miRNAs in PE pathogenesis is not completely known. We document known information on dysregulation of MicroRNA in placenta linked to preeclampsia in the development of biological tools for diagnosis, treatment and prevention. Detailed functional analysis of these differentially expressed placenta specific miRNAs wills us to better understand the nature of PE, and hopefully, contribute to the early detection of this syndrome. The future functional research including in-vitro and in-vivo approaches of the biological pathway of placental specific miRNAs may enhance our understanding of the pathogen¬esis of PE and aid in the development of new therapeutic strategies.

## Figures and Tables

**Figure 1:**
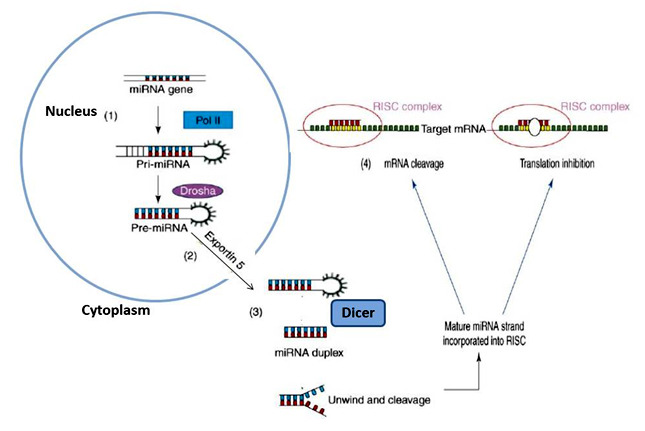
Biogenesis and function of miRNAs. (1)The miRNA biosynthesis begins with RNA polymerase II-dependent transcription of pri-miRNAs from miRNA genes that reside in the introns of their host genes; (2) Pri-miRNAs fold into distinctive stem-loop precursors. In mammals, the long pri-miRNAs are first processed in the nucleus by a microprocessor complex that is composed of the RNase III endonuclease Drosha and a dsRNA-binding protein DGCR8, forming 60–70 nucleotide pre-miRNAs with stem-loop structure and 30 overhangs. The pre-miRNAs are then exported from the nucleus into the cytoplasm by Exp 5; (3) and further processed by another RNase III enzyme, Dicer, into miRNA duplexes. (4) Finally, the miRNA duplex is unwound; one strand functions as the mature miRNA, which is incorporated into the RISC complex [131].
